# Hurricane-Induced Rainfall is a Stronger Predictor of Tropical Forest Damage in Puerto Rico Than Maximum Wind Speeds

**DOI:** 10.1038/s41598-020-61164-2

**Published:** 2020-03-09

**Authors:** Jazlynn Hall, Robert Muscarella, Andrew Quebbeman, Gabriel Arellano, Jill Thompson, Jess K. Zimmerman, María Uriarte

**Affiliations:** 1 Department of Ecology, Evolution and Environmental Biology, Columbia University, New York, NY USA; 2 Department of Plant Ecology and Evolution, Uppsala University, Uppsala, Sweden; 3 Ecology and Evolutionary Biology, University of Michigan, Ann Arbor, Michigan USA; 4 ForestGEO, Smithsonian Tropical Research Institute, Washington DC, USA; 5 Centre for Ecology & Hydrology, Bush Estate, Penicuik, Midlothian EH26 0QB UK; 6 Department of Environmental Sciences, Universidad de Puerto Rico, San Juan, Puerto Rico

**Keywords:** Forest ecology, Tropical ecology, Ecology, Environmental sciences, Natural hazards

## Abstract

Projected increases in cyclonic storm intensity under a warming climate will have profound effects on forests, potentially changing these ecosystems from carbon sinks to sources. Forecasting storm impacts on these ecosystems requires consideration of risk factors associated with storm meteorology, landscape structure, and forest attributes. Here we evaluate risk factors associated with damage severity caused by Hurricanes María and Irma across Puerto Rican forests. Using field and remote sensing data, total forest aboveground biomass (AGB) lost to the storms was estimated at 10.44 (±2.33) Tg, ca. 23% of island-wide pre-hurricane forest AGB. Storm-related rainfall was a stronger predictor of forest damage than maximum wind speeds. Soil water storage capacity was also an important risk factor, corroborating the influence of rainfall on forest damage. Expected increases of 20% in hurricane-associated rainfall in the North Atlantic highlight the need to consider how such shifts, together with high speed winds, will affect terrestrial ecosystems.

## Introduction

Cyclonic storms (hurricanes, typhoons, and cyclones) represent the dominant natural disturbance for many coastal forests^1–4^. Multiple lines of evidence indicate that atmospheric warming will lead to more intense tropical cyclones^5^. Sea surface temperature increases in most regions of tropical cyclone formation suggest that maximum wind speeds will rise and storms are likely to intensify more rapidly^6^. Anthropogenic warming will also lead to higher atmosphere moisture content and increases in tropical-cyclone rainfall rates^6–9^. Increases in the intensity and frequency of tropical cyclones may reduce the ability of tropical forests to sequester carbon^10^.

Tropical forests account for ~70% of the gross carbon sink in the world forests (~4.0 Pg C year^−1^)^11^. Although land use change is the predominant driver of change in the tropical forest carbon sink, natural disturbance (e.g., fires, cyclonic storms) can also have important effects^10,12^. Models and empirical evidence agree that ecosystems are generally carbon sources immediately following disturbance, but are likely to shift to carbon sinks as vegetation recovers^10,13,14^. Forecasting the impacts of a greater number of more severe storms on the ability of tropical forests to act as a carbon sink requires consideration of myriad risk factors that determine the magnitude of storm impacts on vegetation across landscapes.

Observational and modelling studies suggest that forests growing at high elevations or on windward slopes are more exposed to high wind speeds, and experience greater damage and tree mortality from severe storms^1,15–18^. Associations between topography and tree damage may also be mediated by geology and soil characteristics^19^. Restricted root growth of trees growing on ridges, in shallow soils, or soils with poor drainage, may make trees more vulnerable to wind-throw and stem break^1,15,16^, particularly when extreme winds are accompanied by large amounts of rainfall and flooding^20^. Forest stand attributes are also significant predictors of tree damage and mortality as tall forests and dense canopies are generally more susceptible to wind damage^1,21,22^.

Detecting landscape scale effects of cyclonic storms causing forest damage is difficult using a field sampling approach. Extreme wind speeds are highly patchy^23,24^, and the effect of large-scale factors, such as spatial variation in storm meteorology and landscape heterogeneity on tree damage and mortality are difficult to detect. When based on field plot observations, assessments may miss up to 17% of mortality^25^ and small field plot studies lack the statistical power needed to assess drivers of vegetation damage^26,27^. Remote sensing approaches allow estimation of forest damage across large areas and identification of risk factors that lead to spatial heterogeneity in storm impacts.

On September 6, 2017 Hurricane Irma passed roughly 100 km off the northeast coast of Puerto Rico bringing over 300 mm of rain to some parts of the island, but generally there was little damage to the forests. Two weeks later, on September 20, 2017, Hurricane María caused widespread damage to forests when it made landfall on the island with maximum sustained wind speeds as high as 210 km hr^−1^ and in some areas, nearly 1,500 mm of storm-related rainfall over 48 hours (Supplementary Fig. S1).

**Figure 1:**
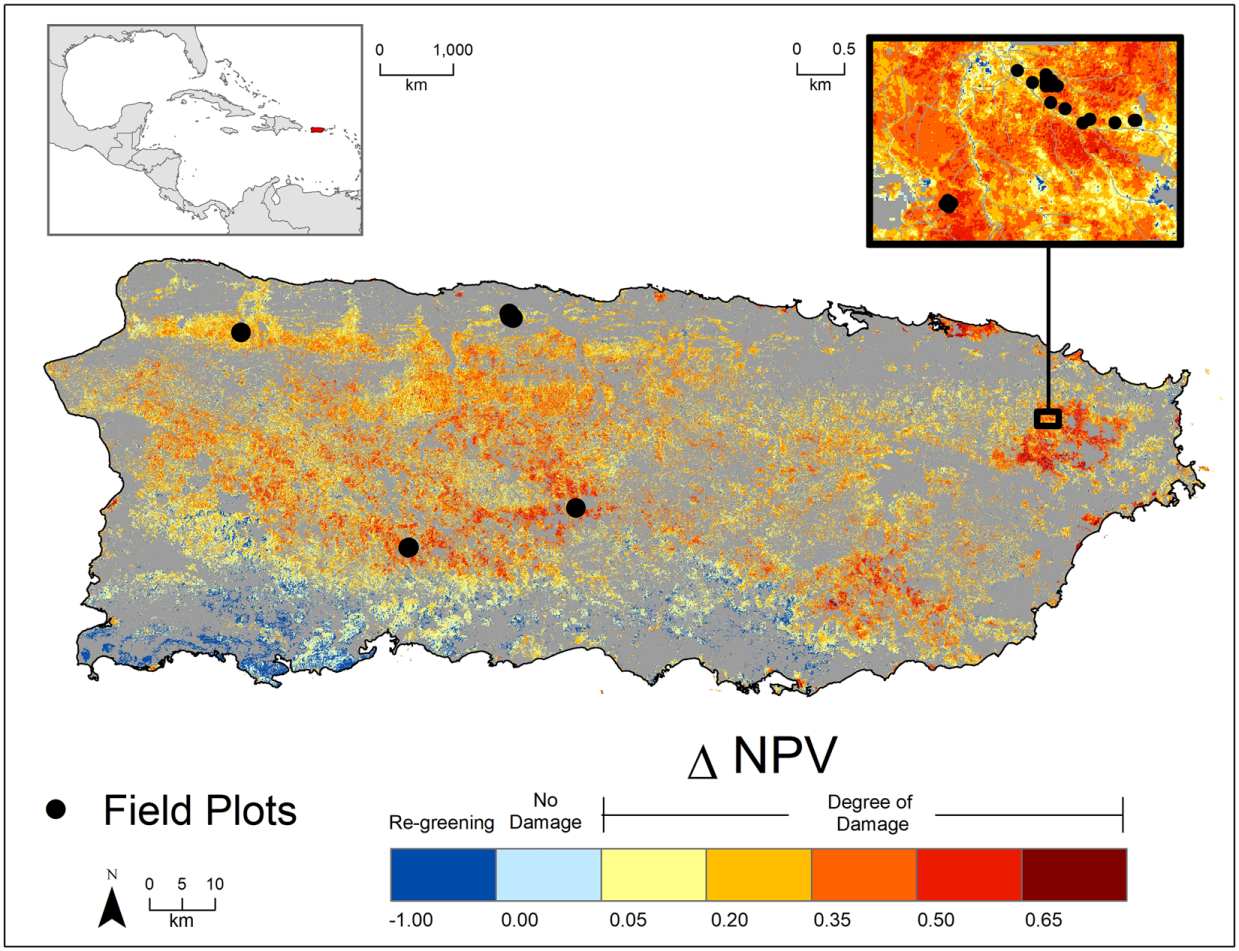
Fractional change in non-photosynthetic vegetation (ΔNPV) in forested areas of Puerto Rico caused by Hurricanes Irma and María in September 2017. Blue colours indicate absence of damage, including re-greening, while tan to dark red colours indicate damage. A value of 0–0.05 (i.e. light blue) indicates little or no change from the year before the storms (September 15 – November 1, 2016) to after the storms (September 21-November 1, 2017). Black circles indicate locations of field plots measured for this study. The additional inset panel shows the locations of the plots clustered within the boundaries of El Yunque National Forest. Clustered plots include subsections of the Luquillo Forest Dynamics Plot, all subsections of a forest succession plot, all elevation gradient plots, and two climate gradient plots (Supplementary Table S1). Non-forested areas and forested areas not included in the study area due to cloud cover for the study period are shown in grey. ΔNPV was generated from Sentinel-2 TOA imagery^28^ in Google Earth Engine^74^ and visualized using ESRI ArcGIS software (version 10.5 https://desktop.arcgis.com/en/arcmap/).

Here, we combine field observations and data from remotely-sensed images to estimate total aboveground biomass (AGB) lost to Hurricanes Irma and María in Puerto Rican forests. We then examined the importance of meteorological, landscape, and forest stand-level risk factors that influenced spatial heterogeneity in forest damage. Forest damage (proportion of aboveground biomass lost) was quantified using 25 forest inventory plots from mature, old-growth forests distributed across Puerto Rico (Fig. 1, Supplementary Table S1). Field damage estimates were used to calibrate a remotely sensed index of damage derived from cloud-free composites of Sentinel-2^28^ top-of-atmosphere reflectance image that show the fractional change in non-photosynthetic vegetation (ΔNPV) over time across Puerto Rico (312,319 ha study area). To control for forest seasonal phenology, the images used were collected the year before Hurricanes Irma and María (September 15-November 1, 2016) and soon after the two hurricanes (September 21-November 1, 2017). This method (ΔNPV) uses spectral unmixing to determine the change in NPV (i.e., woody tree stems and branches) visible from the satellite. Such change is a reasonable indicator of forest damage from cyclonic storms and wind disturbance^29–32^. We evaluated the relative importance of meteorological, landscape, and stand-level risk factors for forest damage using a random forest model^33^. Meteorological risk factors include two-week antecedent rainfall prior to Hurricane María (including that from Hurricane Irma), Hurricane María maximum sustained one-minute winds speeds, and total Hurricane María-related rainfall. Landscape risk factors included topographic indices (i.e. slope, curvature, and wind exposure to Hurricane María), surface-level geological substrate, and soil water storage. Canopy height was used to assess the impact of forest stand structure on damage.

## Results

### Field damage assessment and landscape aboveground biomass (AGB) estimation

**Figure 2:**
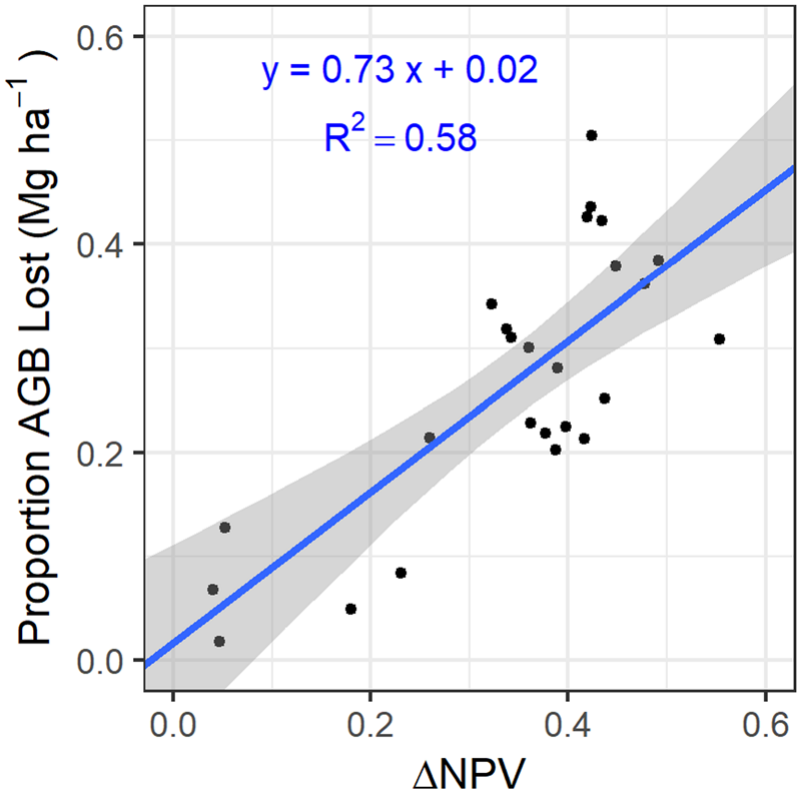
Results from the linear regression between the change in non-photosynthetic vegetation (ΔNPV) and the proportion of aboveground biomass (AGB, Mg ha^−1^) lost as a result of Hurricanes María and Irma, as estimated from the 25 field plots. As a negative ΔNPV value indicates regreening after the hurricanes and no field plots displayed negative ΔNPV values, the figure x-axis (ΔNPV) lower limit was set to zero for plotting purposes. Grey areas indicate 95% confidence intervals for the slope. AGB lost was estimated by including the stem-level AGB for all stems broken, uprooted, or presumed dead, as well as the estimated branch AGB lost based on canopy surveys. All leaf AGB was considered lost (see Supplementary Information for alternative AGB loss estimates).

**Figure 3:**
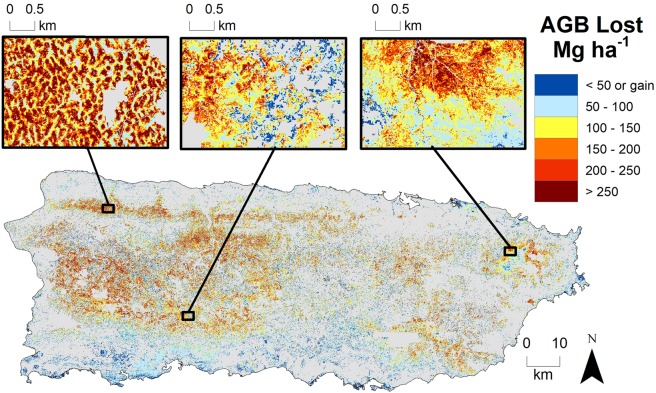
Estimated AGB lost as a result of Hurricanes Irma and María. AGB loss (Mg ha^−1^) estimates were calculated by multiplying the estimated proportion of AGB lost to the hurricanes (Fig. 2) by the estimated pre-hurricane AGB (Supplementary Fig S8). Estimates for AGB were calculated using a regression between field inventory data and area-weighted LiDAR-derived canopy height for field plots. Estimated AGB loss is shown in colours from blue to red, representing low to high values of damage, respectively. Three insets are shown to highlight the variation in damage patterns around the island. Areas with no data are shown in grey. This figure was generated using Sentinel-2 imagery^28^ in Google Earth Engine^74^, canopy height derived from USGS 3DEP and field plots and visualized using ESRI ArcGIS software (version 10.5 https://desktop.arcgis.com/en/arcmap/).

Forest damage quantified using ΔNPV was highly variable across the 312,319-ha study area (Fig. 1), although nearly all (ca. 92%) pixels experienced some degree of damage (Supplementary Fig. S2). Values of ΔNPV were positively associated with all field-plot based estimates of damage (Supplementary Fig. S3). Among the plot damage metrics considered, the proportion of aboveground biomass (AGB) lost to the storm had a strong relationship with ΔNPV (R^2^ = 0.58) (Fig. 2). Total pre-hurricane AGB for the study area was 45.87 Tg (95% CI = 33.29, 65.27) and an estimated 23% of standing pre-storm AGB was lost to the storms in forests across the island (10.44 Tg, CI = 8.11, 12.77). This damage translated to a total loss of 5.22 Tg (CI = 4.06, 6.38) of carbon from broken, uprooted, and dead stems, as well as estimated tree-level branch and leaf loss of standing stems (Fig. 3).

Since we considered all stem-level AGB of broken trees to be lost as a result of the hurricanes, our evaluation overestimates the proportion of pre-storm AGB lost in the field. A complementary, more conservative analysis using 19 plots for which we had information for height to break estimated 20.73% AGB loss at the plot level, 9.67% less than the 30.40% estimated AGB lost for the same 19 plots using our current method. Analyses using the conservative calculations in these 19 plots instead of using the non-conservative estimations in all 25 plots yield an island-level loss of 8.11 (CI = 5.86, 10.37) Tg instead of 10.44 Tg. This difference in estimates indicates that our non-conservative approach may overestimate AGB loss by as much as 2.33 Tg AGB across the study area, approximately 5% of total pre-storm AGB (Supplementary Fig S4).

### Risk factor importance

**Figure 4:**
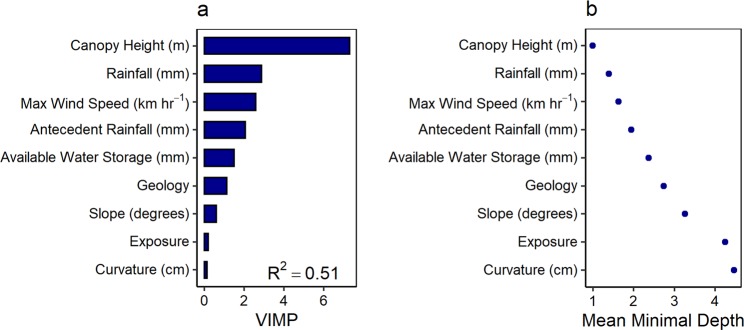
Importance of risk factors from in random forest models (R^2^ = 0.51) in predicting ΔNPV. The importance was determined by randomly permuting values for each predictor variable (VIMP) (a) and by averaging the depth of the first split within the regression tree for each variable across 1,000 regression trees (MD) (b). VIMP is expressed as the difference between MSE of ΔNPV when the risk factor values are permuted, multiplied by 1,000.

Storm meteorological characteristics, landscape structure, and forest stand-level factors all influenced spatial heterogeneity of damage (Fig. 4). Together, these factors accounted for 51% of the observed variation in ΔNPV across the island’s forests. Pairwise correlations between risk factors was generally low, with moderate correlation between Hurricane María storm-related rainfall total and maximum sustained wind speeds for sample locations in this study (Pearson’s r = 0.43, n = 18,903, Supplementary Fig. S1, S5). Risk factor importance was calculated (i) as the impact on the model MSE after randomly permuting each risk factor (i.e. variable importance, VIMP) (Fig. 4a) and (ii) by averaging the depth of the first regression tree split (i.e. minimal depth, MD) for each risk factor across all regression trees for each variable (Fig. 4b)^34,35^.

VIMP and MD methods had nearly identical rankings for risk factor importance. Maximum canopy height was the most important predictor of damage (Fig. 4). Total rainfall and maximum sustained one-minute wind speeds associated with Hurricane María were the next most important risk factors. Antecedent rainfall over the two weeks prior to Hurricane María (including rainfall from Hurricane Irma), and soil water storage capacity (within the top 150 cm of soil), were the next most important factors, followed by geological substrate (at or near-surface) and slope. Curvature and topographic exposure to wind had the weakest association with ΔNPV compared to the other risk factors considered. The strength of interactions between rainfall and risk factors closely tied to soil water status is also indicative of rainfall playing a large role in mediating forest damage by other factors. In contrast, wind speeds displayed few interactions with other risk factors (Supplementary Fig. S6).

Partial dependence analyses of the risk factors indicate that taller forests and areas that experienced high rainfall during the two weeks before (including Hurricane Irma) and during Hurricane María had higher ΔNPV values (Supplementary Fig. S7). A positive relationship between high soil water storage capacity and ΔNPV also suggests that forests in areas with waterlogged soils may be particularly prone to damage. Water logging may have been exacerbated by wet antecedent soil moisture conditions arising from rainfall in the two weeks preceding Hurricane María, including rainfall associated with Hurricane Irma. Forest damage increased linearly with maximum wind speed until 120 km hr^−1^. Forests growing on steep slopes and in valleys subject to flooding had higher ΔNPV values, illustrating that forest growing in these topographic positions are more vulnerable to rainfall damage. Geological substrate had little discernible effect on ΔNPV variation among groups after controlling for the effects of other predictors (Supplementary Fig. S7). Surprisingly, forest growing in topographically wind-exposed areas had similar ΔNPV values to those in areas whose orientation and exposure were expected to have more protection from the hurricane wind (Supplementary Fig. S7).

## Discussion

As in many other tropical regions, Puerto Rican forests experience large catastrophic cyclonic windstorms relatively often. Although there is some variation across the island, return intervals for hurricanes of the severity of the three most recent storms (Saffir Simpson scale 3 for Hugo and Georges and category 4 for H. Maria) are 15–50 and 50–150 years, respectively^17^. Tree species are adapted to these disturbances and tree mortality rates after a severe wind storm tend to be low, ranging between 7–12%, only 2–3 times background mortality rates^22,36–40^. Nevertheless, wetter and more severe storms, such as Hurricane María, expected as a result of global warming will likely exert stronger pressures on forests^41^. Our results suggest that storm meteorological characteristics are a key driver of forest damage, highlighting the need for understanding how more severe storms will influence forest structure, composition, and carbon storage potential.

Beyond any effects of a single storm on forests, our results also demonstrate that not only is the severity of hurricane disturbance key in driving forest damage, but the temporal pattern of disturbance also plays an important role. Trees affected by Irma may have been particularly vulnerable to damage from Hurricane María if stressed and weakened stems, saturating soils and loosened roots make stems more prone to breakage and uprooting^20^, or through other mechanisms of damage accumulation. Our study supports the idea that compounded disturbances can interact in ways that cannot be predicted from the study of a single disturbance^42,43^, an issue ecologists must address to successfully forecast the future of the biosphere under changing disturbance regimes^44^.

The majority of studies on impacts of hurricanes on ecosystems have been conducted at a plot level^4,18,41,45^ or over a limited range of environmental conditions^29,46^. The few studies that have characterized forest damage over heterogeneous environmental conditions using remote sensing^47–49^, have examined a limited set of risk factors, and fewer than a third of studies of this kind have been validated with field data^50^. As a result, our understanding of how expected shifts in storm meteorology may interact with landscape and forest stand attributes to mediate ecosystem vulnerability to hurricanes is extremely limited.

Our analysis integrated field-based estimates of aboveground forest damage with a remotely-sensed damage index to quantify the relative importance of meteorological, topographic, and stand level risk factors on forest damage during Hurricanes María and Irma across Puerto Rico. The few previous landscape and stand level studies on impacts of cyclonic storms on forests^1,22,30,51–55^ have focused on wind as the most important meteorological driver of forest damage. Our study indicates, however, that extremely high rainfall associated with severe hurricanes can be a stronger predictor of forest damage. The importance of rainfall in relation to wind speed is supported by high damage observed in areas with high available water storage and high antecedent rainfall in addition to the failure of topography to protect against damage from extreme winds. Moderate correlations between Hurricane María storm-related rainfall totals and maximum sustained wind speeds for sample locations in this study shows that the two variables display distinct patterns across the island, which in future analyses should be considered both independently and interactively as predictors of forest damage.

High rainfall together with hurricane-force winds can cause uprooting of large trees since roots are more easily dislodged from wet soils^54,56^ and wet foliage increases the weight of the tree crowns. This effect can be exacerbated when soils hold more water or are saturated from antecedent rainfall^1,22,57,58^. Our finding that forest damage was greater in forests growing in areas with high antecedent rainfall and greater soil water storage capacity underscores the importance of rainfall as a key driver of damage. In a storm with extremely high rates of rainfall, such as Hurricane María, the combined effects of rain on already saturated soils and dynamic loading from wind gusts as high as 210 km hr^−1^ likely led to observed island-wide patterns of vegetation damage^59^. Topographic exposure was the least significant risk factor of those examined in this study. This is surprising given the significance of wind speed has in determining damage. One explanation for this may be that the protective role of topography may be lessened in storms of Hurricane María’s magnitude, which may foreshadow similar effects in future intense storms.

The record-breaking rainfall brought by Hurricane María to much of Puerto Rico also led to an unprecedented number of landslides and widespread flooding^59–61^. Landslide occurrence in Puerto Rico is strongly linked to rainfall intensity and duration^62^. Hurricane María was a high-intensity and prolonged rainfall event that caused a landslide density of at least one per km^2^ in 34% of Puerto Rico^47^. Although there were no major landslides in our field plots, the high landslide frequency across the island may have contributed to the relationship between rainfall and satellite estimates of forest damage used in this study, particularly for areas with vulnerable clay-rich soils such as those from volcanic geological substrate (Supplementary Fig. S1)^47^.

Observations and models in previous studies have demonstrated that atmospheric warming will increase rainfall rates as well as wind speed during hurricanes^7^. Some of the most significant increases in cyclone-associated rainfall are likely to occur in the North Atlantic. Models predict that by 2100, maximum sustained wind speeds during tropical cyclones in this region will increase by 6–15%, coupled with increases of ca. +20% in precipitation within 100 km of the storm centre^5,6^. María is the strongest hurricane to have made direct landfall in Puerto Rico since Hurricane San Felipe in 1928^17^ and may presage what atmospheric warming will mean for future North Atlantic hurricanes. Recent analyses^60^ of associations between extreme rainfall events in Puerto Rico and long-term (from 1956–2016) climatic trends in sea surface temperatures found that the probability of observing a storm with precipitation similar to that of Hurricane María has increased almost five times, and hurricane return intervals have decreased by 50%. Expected change in cyclonic storm meteorology and our results suggest the impacts of rainfall on forest damage deserve more research attention.

## Methods

### Study site

Tropical forests in Puerto Rico are an ideal system to examine meteorological, landscape, and forest stand-level risk factors associated with severe hurricanes, as the island encompasses a variety of marked environmental gradients in a relatively small area (910,400 ha). The island’s complex geologic history is reflected in its rugged topography (0–1,338 m a.s.l.) and diverse parent soil materials, which include alluvial, limestone, volcanic, and ultramafic materials^63^ (Supplementary Fig. S1). Mean annual precipitation, ranges from 800 to 4,500 mm yr^−1^^64^. For this study, we limited our study area (312,319 ha) to forested areas in Puerto Rico^65–67^. Field plots^68–71^ cover the rainfall distribution, elevation gradients and two dominant geologies (volcanic and limestone), that are reflected in the diversity of forest characteristics present in Puerto Rico (Fig. 1, Supplementary Fig. S1, Table S1).

This study considers the impacts of Hurricane Irma, a category 5 storm that passed 100 Km to the northeast coast of Puerto Rico on September 6^th^, 2017 together with Hurricane María, a category 4 storm which crossed Puerto Rico on September 20^th^, 2017. Since these storms occurred within 15 days of one another, it is impossible to differentiate the effects of each Hurricane on forest damage. However, the majority of tree damage is ascribed to Hurricane María based on the visible effects of each hurricane on the forests (author observations, 2017) and the objective measures of storm intensity in Puerto Rico (see Supplementary Methods).

### Image processing

To assess damage with remote sensing, Sentinel-2 TOA reflectance images^28^ for Puerto Rico were collected from September 15 - November 1, 2016 (the year before Hurricane María) and September 21 - November 1, 2017 (after Hurricane María) (see Supplementary Methods). Images were selected for the same months of each year (2016 and 2017) to represent the before and after periods to control for seasonal phenology. Pixels without cloud or cloud shadow were retained from the original Sentinel-2 TOA images within the study period (see Supplementary Methods), and used to create composite cloud-free images for the selected periods before and after the hurricanes, using the median non-masked pixel for each pixel location, within each series of images. Primary and secondary roads in the forest area were masked using TIGER 2016 (U.S. Census Bureau, 2016)^72^, and streams were masked using a stream file taken from the Puerto Rico Center for Municipal Revenues (http://gis.otg.pr.gov)^73^. Image processing was conducted using Google Earth Engine^74^ and ESRI ArcGIS software (version 10.5 https://desktop.arcgis.com/en/arcmap/).

### Spectral mixture analysis

We used spectral mixture analysis (SMA) to quantify the pixel-level change in non-photosynthetic vegetation (NPV) fraction between composite images to obtain our metric of storm damage. SMA assumes that every pixel is a linear combination of target endmember spectra (i.e. green vegetation, shade, and NPV), and quantifies the per-pixel fraction of each endmember^75^. As storm damage increases the amount of woody and dead vegetation exposed to the sensor, the increase in NPV fraction (ΔNPV) after Hurricane María reflects the magnitude of storm damage^29–32^.

We applied linear spectral unmixing to each composite image using endmembers for green vegetation (GV), NPV, and shade. Endmember values were determined following similar analyses conducted in the Amazon^31,32^. Following unmixing, we normalized the fraction of NPV without shade as NPV/(GV + NPV) so that fractions reflect only relative proportions of NPV and GV, and not differences due to effects of shading^75^. ΔNPV was calculated by subtracting the normalized NPV fraction in the post-storm composite from the pre-storm composite. In a previous study in the Amazon^32^, ΔNPV acted as a signal for damage that lasted for approximately one year following an extreme wind event, until post-storm recovery generated sufficient new green tissues (mostly leaves) to obscure the NPV signal. In Puerto Rico, estimated NPV decreased in large areas in the south and other small patches scattered around the rest of the island after the storm, suggesting that these areas suffered minor damage from Hurricane María (Fig. 1, Supplementary Fig. S2). Rapid greening in the dry forests of the southwest portion of the island, far from the storm track, was likely the result of high rainfall without severe wind damage. We set these negative ΔNPV values to zero for the aboveground biomass estimation, as we are interested in estimating vegetation damage, rather than forest re-greening after the storms (Fig. 3).

### Field damage assessment and landscape aboveground biomass (AGB) estimation

To validate our remotely-sensed index of damage (ΔNPV), forest damage was assessed across a series of 25 permanent georeferenced field mapped plots distributed across the island (Fig. 1, Supplementary Table S1)^68–71^. A survey of all trees ≥10 cm in diameter at breast height (DBH: 1.3 m from the ground) was carried out in 23 plots between January and May 2018, and in the remaining 2 plots in the autumn of 2018, to assess tree damage resulting from the hurricane. For this study, trees were marked as dead if they had no living foliage at the time of survey. The survey recorded several qualitative and quantitative observations on tree damage resulting from the hurricanes, including the type of damage to stems (uprooting or break), tree crown damage and branch loss using methods similar to other studies conducted in Puerto Rico^43,76^. Field assessments for loss of branches and canopy damage were combined into three classes: low (0–25% loss), moderate (25–75%), and severe (75–100%). To link field plot data to the Sentinel images required that we aggregate tree-based damage data to the pixel scale (Sentinel-2 pixel size is 10 m). Plots differ in size and shape, so we used two approaches. For small plots (i.e. <1 ha) we aggregated data for the entire plot. For larger plots, we separated data into adjacent non-overlapping subsections (Fig. 1, Supplementary Table S1). The old-growth forested area of the largest plot, the 16 ha Luquillo Forest Dynamics Plot, was divided into six 0.36 ha subplots, while the 1 ha old-growth plot from a set of chronosequence plots was divided into four subplots ranging in size from 0.23 to 0.24 ha. Only old-growth forest plots were considered and two plots that had been assessed for damage were eliminated, one due to cloud shadow and the other for partial cloud cover in the Sentinel-2 composites.

### Pre-hurricane AGB

We calculated AGB (leaf, branch, and stem levels) of stems ≥10 cm in DBH for all dicot tree species present within the 25 plots, using DBH measurements and the allometric equation developed for general dicots in forests in Puerto Rico^77^. This size class accounts for the majority of AGB in the plots. Height for the dominant palm species *Prestoea acuminata* var. *montana*, the most abundant tree species in some forests in the island, was estimated from DBH following the approach outlined in a previous study of palm diameter and height relationships^43^. A separate allometry using estimated palm height^77^ was used to estimate *P acuminata* AGB. Pre-hurricane landscape-level AGB (Mg ha^−1^) for the study area (305,972 ha after removing roads, streams, and cloud-covered pixels) was calculated using a regression of field-estimates of AGB and LiDAR-derived canopy height data from the 3D Elevation Program (3DEP)^78^. The regression accounted for 37% of observed variation in field estimates of AGB (Supplementary Fig. S9). Pre-hurricane canopy height for the entire study area was derived from the 2016 3DEP LiDAR data by subtracting last return elevation values from that of first returns. Errors and unrealistic canopy height values (i.e. <0 m or >40 m) were omitted (Supplementary Fig. S1I). Field canopy height values were estimated as the area-weighted average of canopy height values for pixels within each plot.

#### AGB loss to hurricanes

Direct observations of forest damage were used to estimate the field plot-level AGB damaged from the combined impacts of Hurricanes Irma and María. Plot-level damage was characterized using a variety of metrics including total damaged biomass, proportion of pre-hurricane biomass damaged, proportion of basal area severely damaged (i.e. broken, uprooted or dead = BUD), stem density BUD (≥10 cm DBH), and proportion of stems BUD for each plot. AGB loss was calculated by summing the stem-level biomass of all stems broken, uprooted, or dead. For standing stems, field-based estimates of the proportions of canopy damaged (see above) were multiplied by the allometrically estimated branch-level AGB to estimate AGB lost from branch damage. The middle percentage value for the canopy damage classes described above (i.e. low = 12.5% loss, moderate = 50% loss, and severe = 87.5% loss) were assumed for AGB loss calculations. All leaf AGB was considered lost, based on field observations. Branch and leaf loss of standing, non-BUD stems were added to the estimate of stem AGB loss.

Landscape-level biomass loss was estimated from the regression between ΔNPV and the proportion of estimated AGB lost from field observations in the 25 plots (Fig. 2, Supplementary Fig. S3). For all pixels with damage, the proportion of AGB loss was calculated and multiplied by the estimate of pre-hurricane AGB. Carbon mass was assumed to be 50% of AGB values (Fig. 3)^79^. Estimates and 95% confidence intervals of AGB and AGB lost to the storm were calculated based on the regressions between field-based estimates and remotely sensed estimates for the 25 field plots (Fig. 2, Supplementary Fig. S9). ΔNPV and canopy height values for each pixel in the study area were included in the prediction as new data. The sum of pixel-level estimates and lower and upper confidence intervals represented the AGB and AGB lost for the study area.

Since many broken and uprooted stems will not completely die or will have delayed mortality^41^, this method likely overestimates immediate biomass loss. To quantify the potential error in our estimate of AGB loss, we used the most conservative estimate of AGB loss available with the data that was collected (Supplementary Fig. S4). In some field plots (n = 19), stem break height and the damaged branch percent were recorded. To estimate AGB loss above the stem break, stems in the plots with stem break height were modelled as a series of truncated cones. The radius of each cone was estimated using default parameters of tapering functions^80^, which have been found to be the most effective among several competing models in tropical forests^81^. The proportion of AGB above a given height was estimated, and AGB lost above the break from each stem was extracted from tree-level AGB (see above). In instances where percent branch damage was recorded, this was used to estimate branch damage rather than the midpoint of each damage category (see above). We used estimates of the proportion of AGB lost within the field plots to compare the method considering all broken, uprooted, and dead stem loss as well as branch and leaf loss to this conservative method using estimated AGB above break height and percent branch loss. As we have no basis for deciding which trees will live or die beyond the proportion of mortality of uprooted trees that died after previous hurricanes, both methods considered all uprooted stems living at the time of measurement to be lost, which may overestimate the actual future loss of these stems by as much as 45%^43^. Nevertheless, uprooted stems usually remain alive by sprouting new stems from the fallen trunk while much of the biomass in the main fallen stem will be lost. The landscape-level estimate was recalculated using the regression between plot-level proportion of AGB lost and ΔNPV to consider the total potential error in AGB loss.

### Random forest models: evaluation of risk factor importance

Meteorological, landscape, and stand characteristics were used as risk factors for forest damage (see Supplementary Methods). Meteorological characteristics include Hurricane María maximum 1-minute sustained wind speeds (km hr^−1^) (RMS HWind, https://www.rms.com/models/hwind)^82^, rainfall data (NOAA, https://water.weather.gov/precip/download.php) for the day of Hurricane María landfall in Puerto Rico and the following day (September 20–21, 2017), and antecedent rainfall for the two weeks before Hurricane María (September 6–19, 2017), including that which fell during Hurricane Irma (on September 6^th^). Landscape characteristics include indices of slope and general curvature (curvature in the direction of water flow)^78^, binary topographic wind exposure (NOAA NHC, https://www.nhc.noaa.gov/data/)^17,83^, soil water storage capacity (gSSURGO, (https://gdg.sc.egov.usda.gov/), and bedrock geology type^84^. Canopy height was also included as a stand characteristic^78^.

### Statistical analyses

Fitting models with all our variables is computationally unfeasible for all the pixels in the island. For that reason, we assessed the association between ΔNPV and risk factors by randomly sampling 20,000 points within the study area. Sample points were excluded if we lacked risk factor values for the location, which left a final total of 18,903 ΔNPV observations in the analyses. Risk factor values were extracted for a random subset of ΔNPV pixels using the R package “raster”^85^.

Random forest models were fit to assess the relationship between the sampled ΔNPV and risk factors. Random forest models fit many nonparametric regression-trees to the data using a randomly selected subset of predictors per tree. As output, they give the average prediction of all the individual regression-trees^33^. This process accommodates nonlinear relationships between the response (i.e. ΔNPV) and predictor variables (i.e. risk factors) as well as interactions between variables. All risk factors (i.e. Hurricane María maximum wind speeds, María-related rainfall, two week antecedent rainfall, slope, curvature, topographic exposure to Hurricane María winds, available soil water storage, geological substrate, and canopy height) were included in the model and a random forest model with 1,000 regression-trees was run. Analysis was conducted using the “randomForestSRC” package and the results visualized using the “ggRandomForests” package in R^34,35^. The importance of the individual predictors was assessed using two methods; first, we examined prediction strength while randomly permuting values for each predictor variable (VIMP); second, we calculated the minimal depth for each variable (MD), averaging across the 1,000 regression trees^33,34^. The importance of the selected variables was assessed using both variable importance (VIMP) and minimal depth (MD) methods. Partial dependence plots of the response variable to each risk factor were also generated by averaging prediction values for all other risk factor along the distribution of the risk factor of interest keeping other risk factors at mean values across regression-trees^86^. Partial dependence plots (Supplementary Fig. S7) were created using MD variable sorting importance order (Fig. 4B**)**. We also quantified all pairwise interactions between risk factors in the regression-tree using Friedman’s H factor^87^. This statistic measures how much of the variation of the prediction depends on the interaction among risk factors. It assigns a value of 0 if there is no interaction at all between one risk factor and any others and 1 if all of the variance comes from interactions between risk factors. This analysis was done using the “pre”^88^ and “iml” packages^89^ with a 3,000 point subsample of our 18,903 ΔNPV sample points due to computational constraints (Supplementary Fig. S6).

## Supplementary information

Supplementary Information.
