# Supplementary material for: Hurricane-Induced Rainfall is a Stronger Predictor of Tropical Forest Damage in Puerto Rico Than Maximum Wind Speeds
Source: Sci Rep. 2020 Mar 9;10:4318. doi: 10.1038/s41598-020-61164-2 (PMC7062726; doi:10.1038/s41598-020-61164-2)
Supplement: Supplementary file 1 — Supplementary Information. [file 41598_2020_61164_MOESM1_ESM.pdf]

HURRICANE-INDUCED RAINFALL IS A STRONGER PREDICTOR OF TROPICAL FOREST DAMAGE IN PUERTO RICO THAN MAXIMUM WIND SPEEDS

Supplementary Information

Jazlynn Hall*[1], Robert Muscarella[2], Andrew Quebbeman[1], Gabriel Arellano[3,4], Jill Thompson[5], Jess K. Zimmerman[6], and María Uriarte[1]

[1] Department of Ecology, Evolution and Environmental Biology, Columbia University, New York, NY, USA.

[2] Department of Plant Ecology and Evolution, Uppsala University, Uppsala, Sweden

[3] Ecology and Evolutionary Biology, University of Michigan, Ann Arbor, Michigan, USA.

[4] ForestGEO, Smithsonian Tropical Research Institute, Washington DC, USA.

[5] Centre for Ecology & Hydrology, Bush Estate, Penicuik, Midlothian, EH26 0QB, U.K.

[6] Department of Environmental Sciences, Universidad de Puerto Rico, San Juan, Puerto Rico

*Corresponding author

Methods

Image processing: date selection

To determine dates for pre-hurricane images representative of background forest conditions, we examined patterns in vegetation phenology from 2000 to 2017 using Enhanced Vegetation Index (EVI), a measure of canopy greenness [1], from MODIS Terra Vegetation Index 16-day composites [2]. We chose 2016 to represent pre-hurricane forest conditions as these images capture the most recent pre-hurricane vegetation conditions for our months of interest and there were no outlier values for EVI when compared to values from the years 2010 to 2017 (**Supplementary Fig. S8**). Note that there were no major hurricanes in Puerto Rico between 2016 and 2017.

Image processing: cloud masking

The Sentinel QA60 band was used to mask out clouds and other cloud-affected pixels from all images. However, the Sentinel-2 cloud detection algorithm often poorly predicts cloud cover in the tropics due to complex cloudiness and high water vapor content [3]. Following recommended precautions to further restrict cloud cover thresholds [3], cloud masks were visualized using a B2B3B4 RGB composite display (i.e. a natural colour composite) for images before the hurricanes to find undetected cloud and cloud shadow areas that were missed by the Sentinal-2 algorithm. Band thresholds were determined to implement additional restrictions for pixel values to minimize cloud and cloud shadow. Pixels were classified as cloud if they had higher than expected band values for the aerosol, blue, or red bands (greater than 0.20, 0.17, and 0.20, respectively), which are similar to those from the known cloud areas visualized using the natural composite display. Similarly, pixels were assigned as cloud shadow if they had lower than expected values for the red and vegetation red edge bands (less than 0.03 and 0.18, respectively) [4].

Random forest models: risk factors

Meteorological characteristics

Wind speed. Maximum 1-minute sustained wind speeds (km hr^{-1}) for Hurricane María processed at 1-km resolution were obtained from RMS® HWind (**Supplementary Fig. S1**) (https://www.rms.com/models/hwind). HWind is derived from a series of satellite, aerial, and ground-based observations.

Rainfall. Because saturated soil flooding can exacerbate wind impacts, we also included downscaled rainfall totals during the storm. Storm rainfall data for the day of Hurricane María landfall in Puerto Rico and the following day (September 20-21, 2017) were obtained from NOAA gridded observed precipitation data at approximately 5 km resolution (https://water.weather.gov/precip/download.php) (**Supplementary Fig. S1**). Rainfall estimates are multi-sensor (radar and rain gauge) values from National Weather Service (NWS) River Forecast Centers (RFCs) and mosaicked by National Centers for Environmental Prediction (NCEP). The centre of Hurricane María took 8 hours to cross the island, but areas in Puerto Rico likely experienced tropical storm severity wind speeds and rainfall before Hurricane María made landfall until approximately halfway through September 21 (**Supplementary Fig. S10**). The two-day period used in our analyses includes total rainfall associated with the storm that likely occurred concurrently with at least tropical storm-force winds (63 km hr^{-1}), using storm radii from the National Hurricane Center and Central Pacific Hurricane Center (https://www.nhc.noaa.gov/data/tcr/). Antecedent rainfall data for the two weeks before Hurricane María (September 6-19, 2017), including that which fell during Hurricane Irma (on September 6th), was also included as a proxy for soil moisture conditions at the time of Hurricane María (**Supplementary Fig. S1**).

Landscape characteristics

Topography. To examine the influence of topography on storm impacts, slope and general curvature (curvature in the direction of water flow) were calculated using an island-wide USGS LiDAR-derived

Digital Elevation Model (DEM) with a 5x5m pixel resolution [5]. This LiDAR data was acquired in 2016 and processed using methods outlined by the USGS 3D Elevation Program (3DEP) (https://nationalmap.gov/3DEP/3dep_about.html). There is a DEM data gap in the southeast portion of Puerto Rico, which led to a gap in values for all risk factors calculated using the 3DEP LiDAR values (i.e. curvature, slope, and canopy height). Calculations for curvature (**Supplementary Fig. S1**) and slope (**Supplementary Fig. S1**) were conducted in ArcGIS 10.5 using the 5m DEM and the DEMSurfaceTools extension [6]. Slope was resampled to match the Sentinel-2 ΔNPV 10 m pixel sizes, and general curvature was resampled to 100 m pixel sizes to better account for landscape-level topography. Negative curvature values indicate concave areas (i.e., valleys or low points) where soil water may accumulate, and negative values indicate convex areas (i.e. ridges and high points) where soil water may be lower because of runoff.

Topographic exposure. To reconstruct wind exposure in the island, we used EXPOS, a simple model that combines information on the track a hurricane, wind speeds, and topography to predict heterogeneity in wind impacts across the landscape [7](**Supplementary Fig. S1**). The EXPOS model assumes that wind movement over land decreases sustained wind speeds and increases inflow angles, and then calculates the spatial variation in exposure at the native resolution (5 m) of the DEM. This model has been shown to accurately reconstruct historical exposure to hurricane winds in Puerto Rico at the landscape scale, when compared to historical records [7,8]. Hurricane track data was downloaded from NOAA NHC Tropical Cyclone Best Track archive (https://www.nhc.noaa.gov/data/) and the USGS 3DEP DEM (5 m resolution) was used to estimate exposure [5]. We used a 20-degree inflow angle. EXPOS output is a binary variable indicating areas protected from or exposed to damaging winds.

Soil and geology. To examine the impacts that soil water storage capacity characteristics have on the degree of forest damage, we used water storage capacity (in cm of water) for soil horizons from 0 to 150 cm below the surface (10 m resolution) from the Gridded Soil Survey Geographic (gSSURGO, (https://gdg.sc.egov.usda.gov/). As this data is derived from vector values and transformed into gridded raster pixels, each value is weighted by vector area within the raster pixel (**Supplementary Fig. S1**). The geology of the bedrock that lies at or near the land surface was also considered as a risk factor. Near-surface geology was retrieved from the USGS Generalized Geologic Map of Alaska, Hawaii, Puerto Rico, and the U.S. Virgin Islands [9] (**Supplementary Fig. S1**). Geology was characterized as sedimentary limestone, volcanic, granitic, ultramafic, or quaternary alluvium deposits.

Stand Characteristics

Canopy Height. For the same level of storm exposure, areas with higher canopy and greater biomass are expected to exhibit higher levels of ΔNPV since those areas have more woody material to be damaged [10,11]. High biomass areas tend to have taller trees with large stem diameters and branches. Large branches, have greater drag coefficients and are more likely to be broken off by hurricane winds [12]. Consequently, for the same level of storm exposure, areas with greater biomass are expected to exhibit higher levels of ΔNPV[10,11]. Canopy height was derived from the 2016 USGS 3DEP LIDAR (https://nationalmap.gov/3DEP/3dep_about.html) (**Supplementary Fig. S1**).

References

1. Liu, H. Q. & Huete, A. A feedback based modification of the NDVI to minimize canopy background and atmospheric noise. *IEEE Trans. Geosci. Remote Sens.* **33**, 457–465 (1995).

2. Didan, K. *MOD13Q1 MODIS/Terra Vegetation Indices 16-Day L3 Global 250m SIN Grid V006*. (2015). doi:https://doi.org/10.5067/MODIS/MOD13Q1.006

3. Coluzzi, R., Imbrenda, V., Lanfredi, M. & Simoniello, T. A first assessment of the Sentinel-2 Level 1-C cloud mask product to support informed surface analyses. *Remote Sens. Environ.* **217**, 426–443 (2018).

4. Hollstein, A., Segl, K., Guanter, L., Brell, M. & Enesco, M. Ready-to-use methods for the detection of clouds, cirrus, snow, shadow, water and clear sky pixels in Sentinel-2 MSI images. *Remote Sens.* **8**, 1–18 (2016).

5. Carswell, W. J. *The 3D Elevation Program: summary for Puerto Rico*. (2016). doi:10.3133/fs20133097

6. Jenness, J. DEM Surface Tools for ArcGIS. *May* (2013).

7. Boose, E. R., Foster, D. R. & Fluet, M. Hurricane Impacts to Tropical and Temperate Forest Landscapes. *Ecol. Monogr.* **64**, 369–400 (1994).

8. Boose, E. R., Serrano, M. I. & Foster, D. R. Landscape and regional impacts of hurricanes in Puerto Rico. *Ecol. Monogr.* **74**, 335–352 (2004).

9. Reed, J. C. J. & Bush, C. A. Generalized Geologic Map of Alaska, Hawaii, Puerto Rico, and the U.S. Virgin Islands. (2005).

10. Lugo, A. E. Visible and invisible effects of hurricanes on forest ecosystems: An international review. *Austral Ecol.* **33**, 368–398 (2008).

11. Everham, E. M. & Brokaw, N. V. L. Forest Damage and Recovery from Catastrophic Wind. *Bot. Rev.* **62**, 113–185 (1996).

12. Flynn, D. F. B. *et al.* Hurricane disturbance alters secondary forest recovery in Puerto Rico. *Biotropica* **42**, 149–157 (2010).

Figure S1. Map of risk factors. Meteorological risk factors include (A) maximum sustained (1-minute) wind speeds associated with Hurricane María (RMS® HWind, https://www.rms.com/models/hwind), as well as (B) total storm-related rainfall (Sept. 20-21, 2017) and (C) antecedent and Hurricane Irma-related rainfall (Sept. 6-19) (NOAA, https://water.weather.gov/precip/download.php). Note that the two days during Hurricane María accumulated three times more rainfall than the previous two weeks, Hurricane Irma included. Landscape characteristics considered are topographic (USGS 3DEP, https://nationalmap.gov/3DEP/3dep_about.html), soil (gSSURGO, (https://gdg.sc.egov.usda.gov/), and geological [9] indices including (D) curvature, a measure of convexity of the landscape , (E) slope in degrees, (F) estimated topographic exposure, (G) available soil water storage to 150 cm soil depth, (H) near surface geological substrate. The stand characteristic considered as a risk factor is the (I) LiDAR-derived canopy height (USGS 3DEP, https://nationalmap.gov/3DEP/3dep_about.html). This figure was visualized using ESRI ArcGIS software (version 10.5 https://desktop.arcgis.com/en/arcmap/).

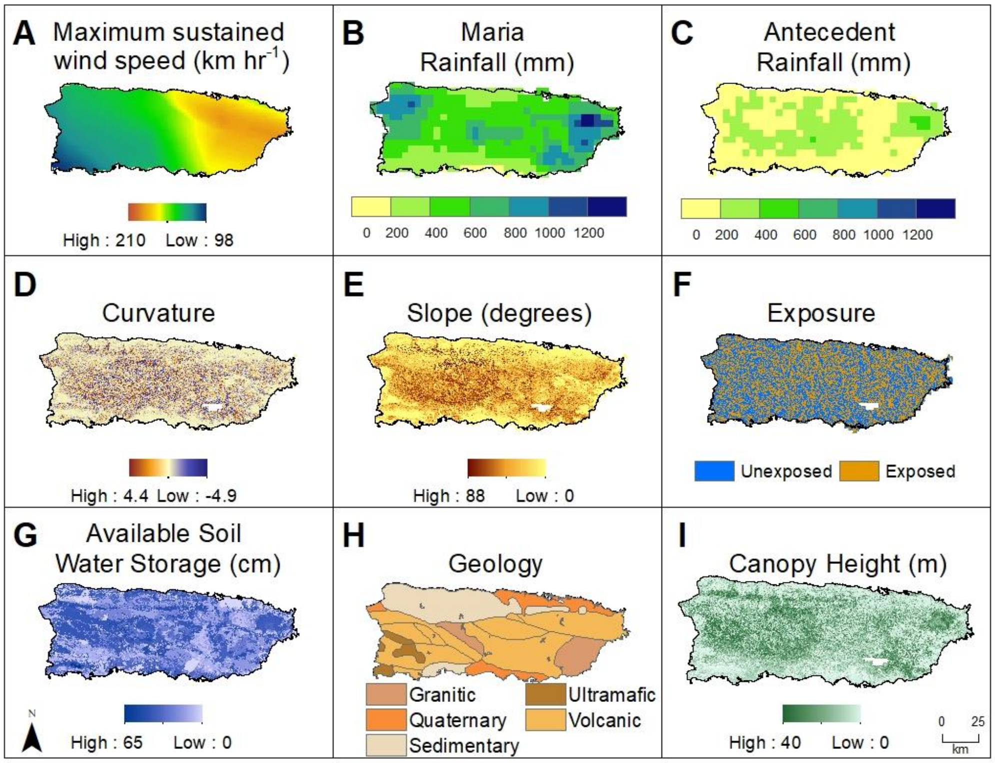

Figure S2. Histogram of ΔNPV values across the study area.

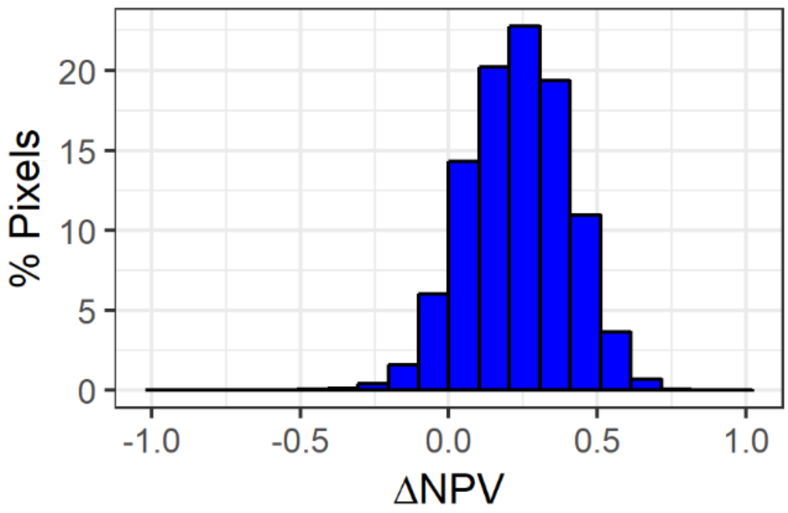

Figure S3. Regressions between alternative field-measured damage metrics (y-axis) and plot-level area-weighted average value of ΔNPV (x-axis) for 25 plots. Grey areas indicate 95% confidence intervals for the slopes. Field damage metrics besides % AGB lost (Figure 2) include (A) log of estimated aboveground biomass (Mg ha^{-1}) lost, including broken, uprooted, or dead stems and branch and leaf AGB lost, (B) percent of basal area damaged that was broken, uprooted, or dead, (C) stem density (stems/ha) of broken, uprooted, or dead stems, (D) and percent of stems broken, uprooted, or dead.

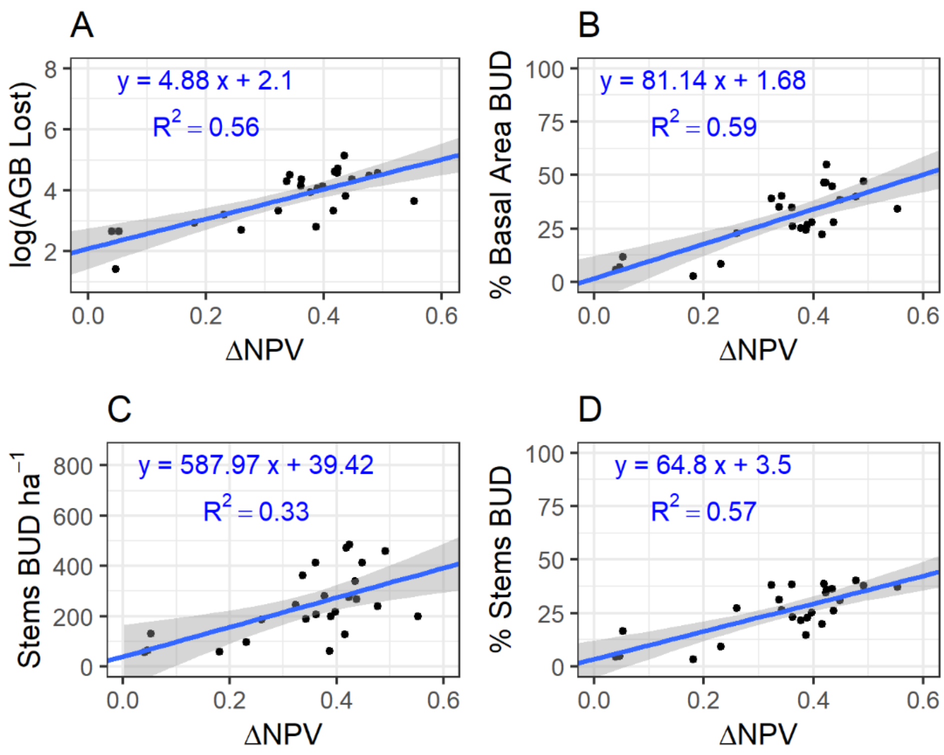

Figure S4. Results from the linear regression between the change in non-photosynthetic vegetation (ΔNPV) and the two estimates of field-measured proportion of aboveground biomass (AGB, Mg ha^{-1}) lost for plots with break height estimates (n = 19). The baseline estimate (blue) considers all AGB from stems broken, uprooted, or dead to be lost as well as AGB loss based on branch damage categories. The conservative estimate (red) considers AGB from uprooted and dead stems lost, in addition to AGB estimated above break height for stems that have break height data (in 19 plots out of 25), and estimated branch damage percentiles.

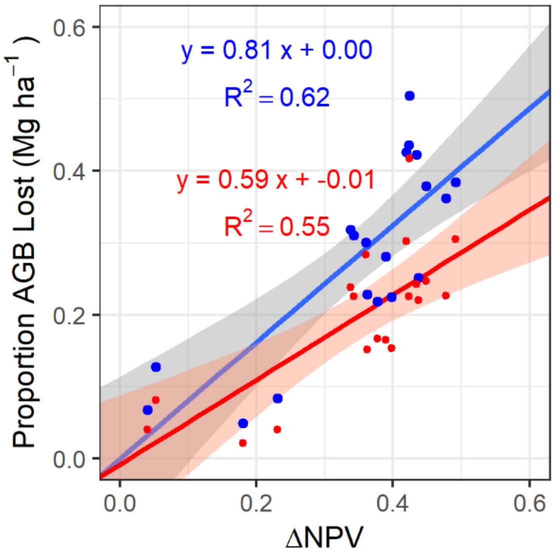

Figure S5. Pairwise linear correlations between ΔNPV and risk factors (Pearson's *r*). Negative correlations are shown in red and positive correlations in blue. White indicates no significant relationship.

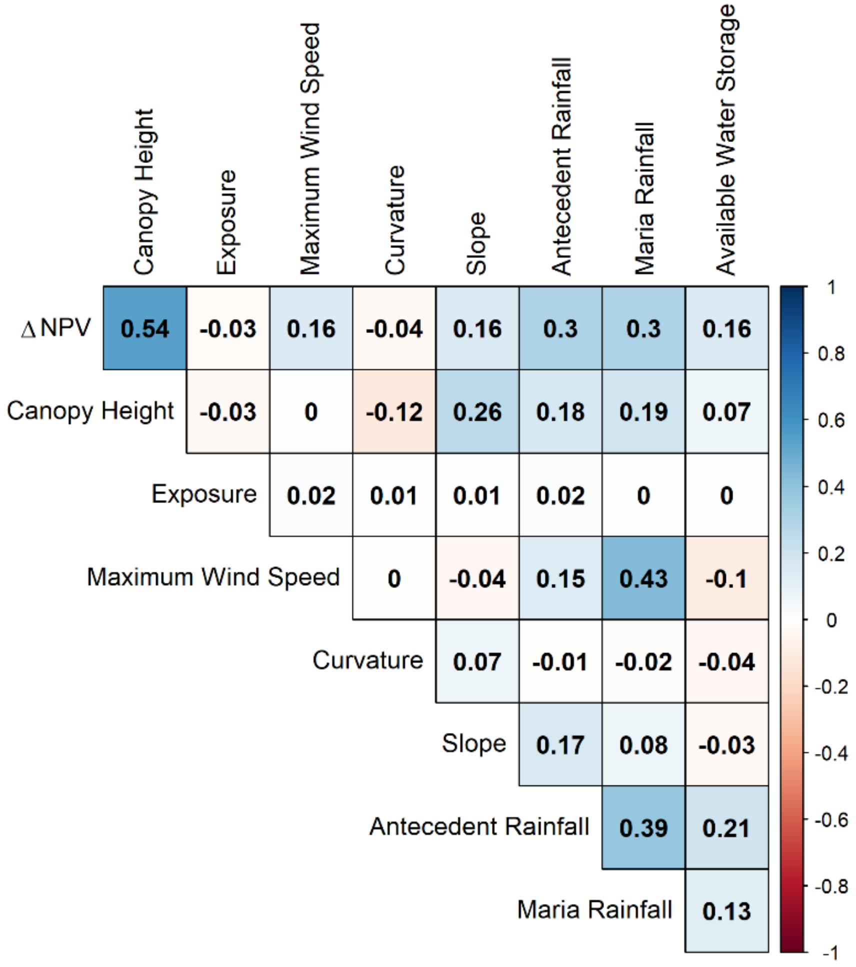

Figure S6. Interaction strength H-factor of risk factors predicting ΔNPV in a subsample of 3000 points across the study area. This is the strength of the interaction between each risk factor and all other risk factors when predicting ΔNPV. The interaction strength indicates the percent of the variance that comes from interactions between a risk factor of interest and the others, where a value of 0 indicates no interactions between a risk factor and any others and a value of 1 occurs when all of the variance can be attributed to interactions between risk factors.

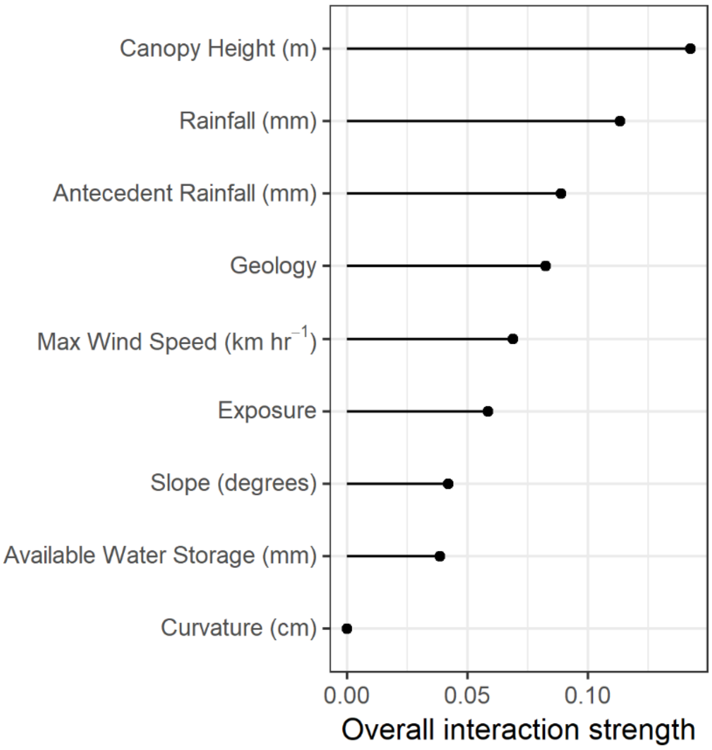

Figure S7 Partial dependence for the variables identified as important for predicting ΔNPV, ordered using the Minimal Depth method. Variables are sorted from most important (A) to least important (I). Partial dependence indicates the predicted response in ΔNPV as a function of each risk factor after controlling for all risk factor effects besides the one of interest. Y-axis limits are the same across rows to better discern individual risk factor effects. Geological substrates are, from left to right: granitic, quaternary alluvium deposits, sedimentary limestone, ultramafic, volcanic. Negative topographic curvature values indicate ridges, positive are valleys, and zero values indicate no topographic curvature. Grey areas for continuous variables display 95% confidence intervals using a loess regression (blue line).

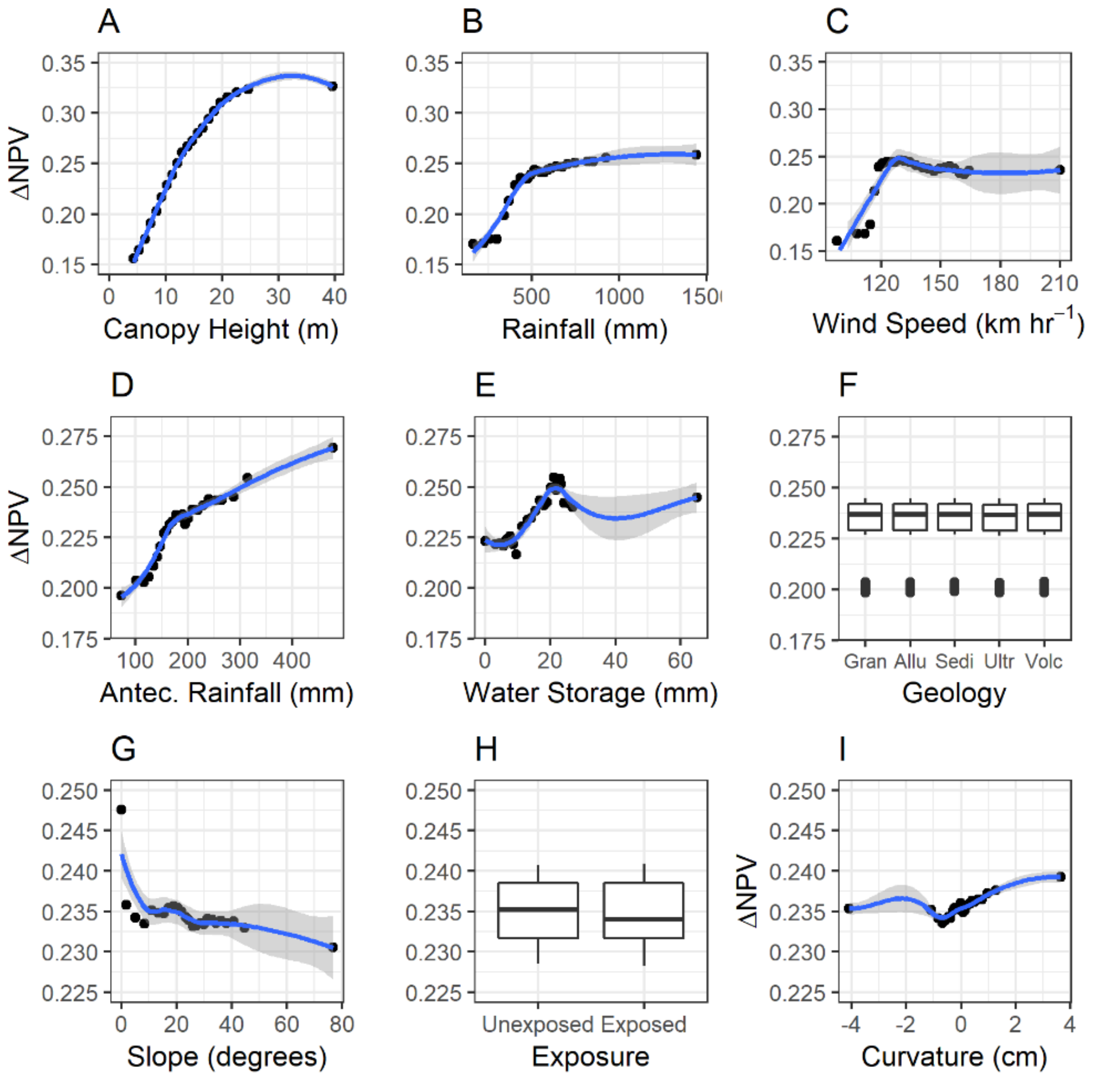

Figure S8. Annual phenology of average enhanced vegetation index (EVI) 16-day composite values for forested areas in Puerto Rico between 2010 and 2017. The blue line shows the chosen pre-hurricane baseline year (2016) and the brown indicates 2017 with a big dip in EVI in September 2017. Grey lines show variation throughout the year for individual years from 2010 through to 2015.

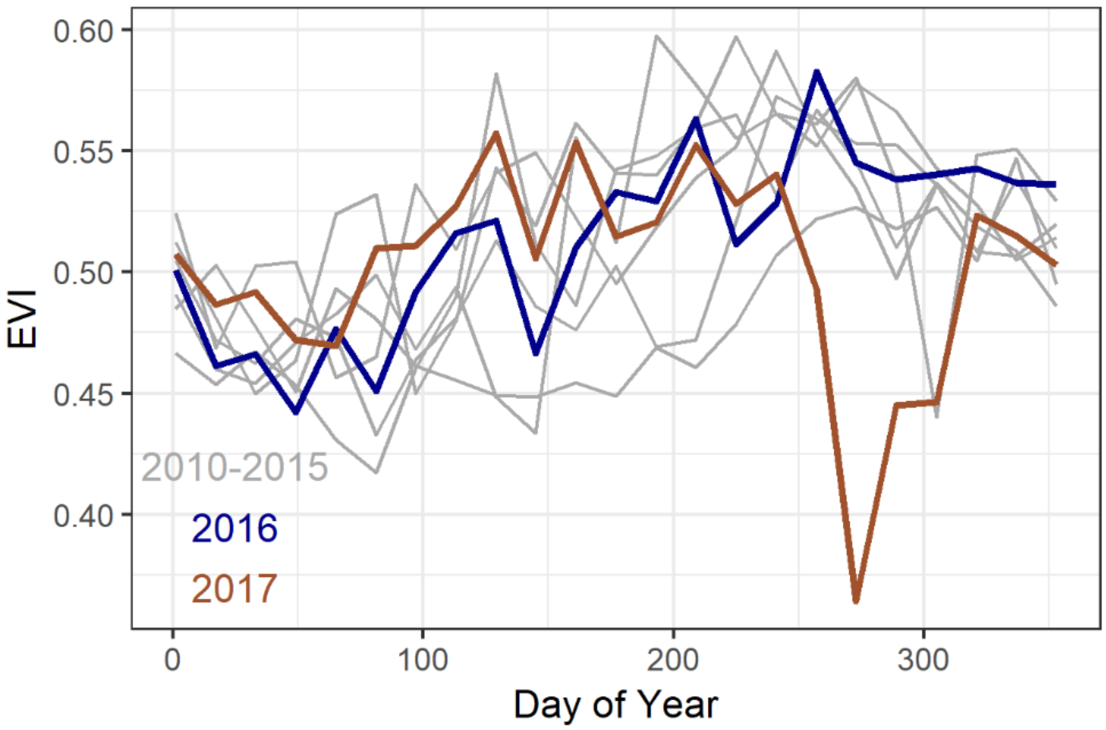

Figure S9. Relationship between plot-level area-weighted values of LiDAR-derived canopy height (m) (x-axis) for pixels within the 25 field plots and the natural logarithm of field-measured AGB (Mg ha^{-1}) before the hurricanes (y-axis) for stems with ≥ 10 cm DBH. Axis limits have been set to show the extent of the field data rather than the origin.

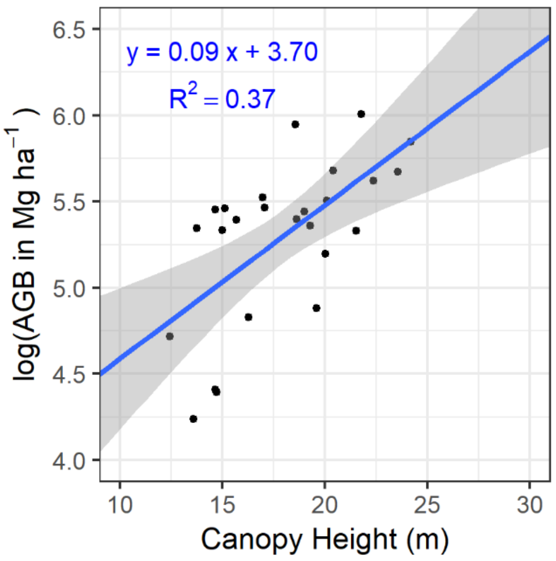

Figure S10. Hurricane María track and radii of spatial extent showing tropical storm-force wind speeds (at least 63 km hr^{-1}) for the first twelve hours of September 21, 2017, taken at 6-hourly intervals (colours distinguish time steps). Figure is based on data from the National Hurricane Center's Tropical Cyclone Reports (https://www.nhc.noaa.gov/data/tcr/). Black arrows indicate Hurricane María direction.

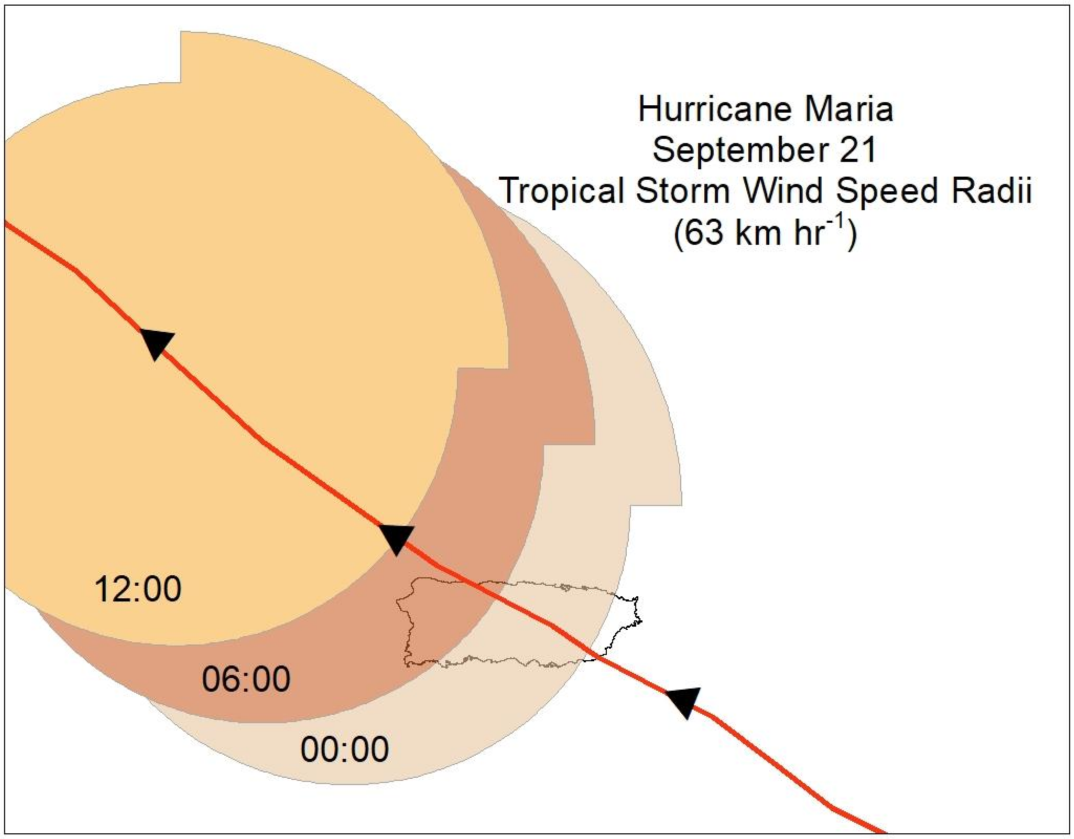

Table S1 Size, number, and physical characteristics of field plots included in the analyses. Original sources for plot data are also included. Some large plots were separated into smaller subplots to make plot area similar across plots for the analysis.

Plot Name	No. plots	Dimensions (m)	Mean rainfall (cm yr^{-1})	Elevation (m asl)	Geology (No. plots)	Source
Elevation Gradient	6	20 x 50	200-400	300-600	Volcanic	Barone et al. (2008)
Forest succession	4	50 x 50	350	167-535	Volcanic	Schwartz et al. (in review)
Luquillo Forest Dynamics Plot	6	60 x 60	350	334-428	Volcanic	Thompson et al. (2002)
Climate gradient	9	50 x 50	150-214	58-945	Volcanic (5) Sedimentary limestone (4)	Muscarella et al. (2016)
